# Supplementary material for: Conserved residues in the wheat (Triticum aestivum) NAM-A1 NAC domain are required for protein binding and when mutated lead to delayed peduncle and flag leaf senescence
Source: BMC Plant Biol. 2019 Sep 18;19:407. doi: 10.1186/s12870-019-2022-5 (PMC6749658; doi:10.1186/s12870-019-2022-5)
Supplement: Supplementary file 2 — Additional file 2: “Supplementary Data”. This file contains supplementary figure and tables referred to in the text. (DOCX 2720 kb) [file 12870_2019_2022_MOESM2_ESM.docx]

**Additional File 2: Supplementary Data**


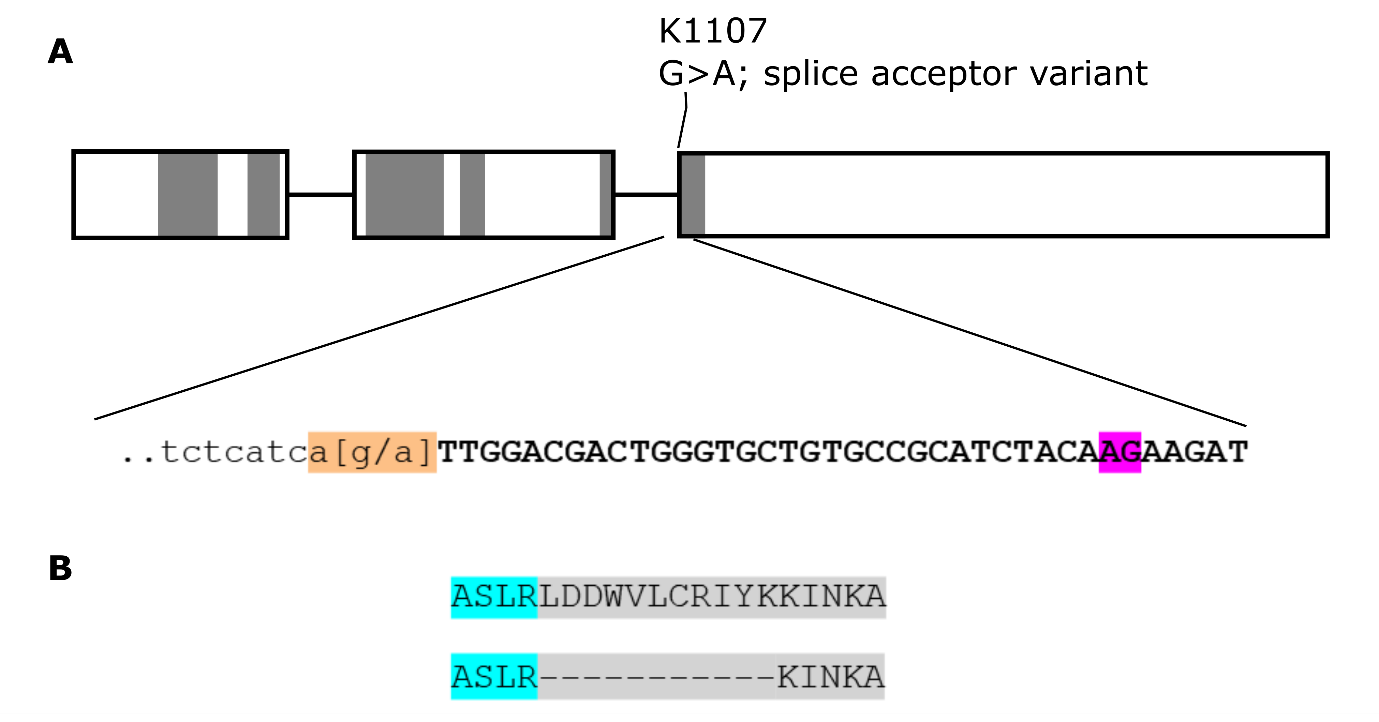


**Figure S1: The splice acceptor variant in K1107 is predicted to lead to an 11-residue deletion.** The Kronos TILLING line K1107 contains a G > A substitution at the splice acceptor site of the third exon (A). This mutation (orange) is predicted to lead to the use of an alternate splice site (purple). This predicted splice isoform contains an 11-residue deletion encompassing the majority of subdomain v (B).


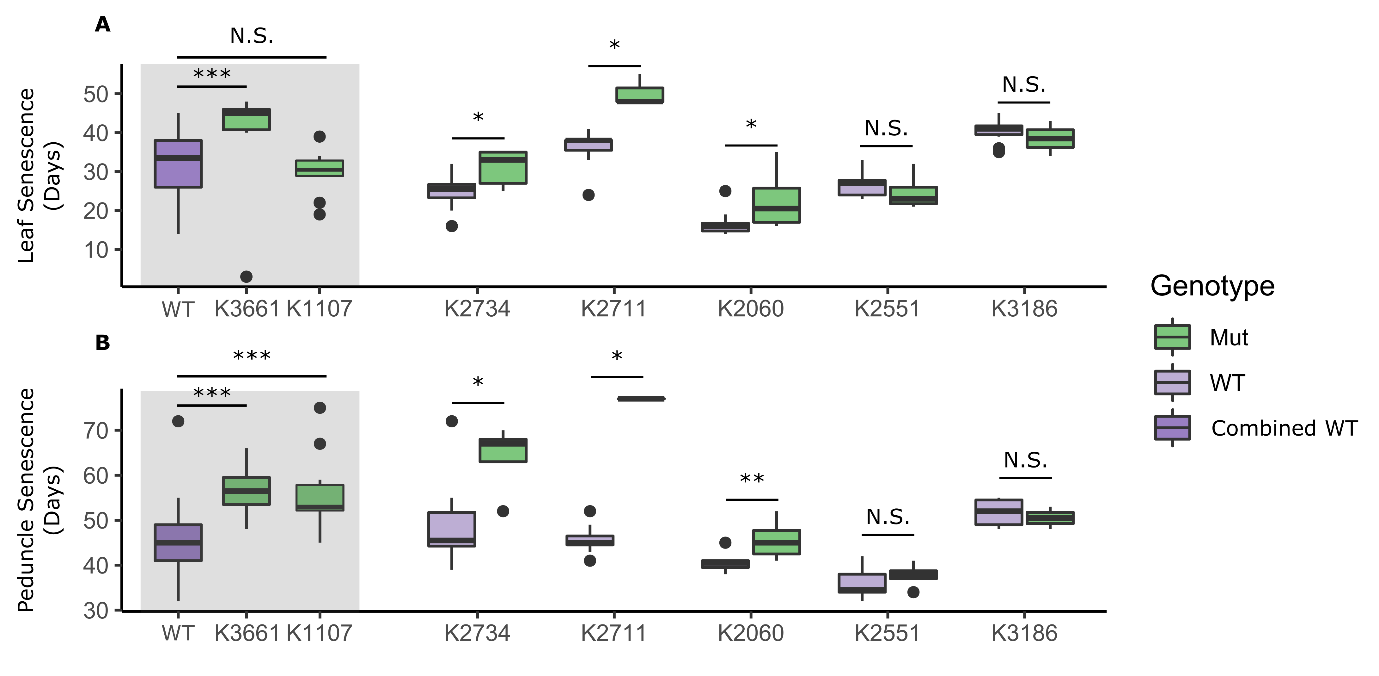


**Figure S2: Lines K2711, K2734, and K3661 are consistently delayed in peduncle senescence.** Initial screening of the TILLING lines, excluding line K2615, found similar results for flag leaf senescence (A) and peduncle senescence (B). Notably, K2060 is also delayed in peduncle senescence in this experiment, and all lines containing missense mutations are slightly delayed in leaf senescence. The remaining mutant lines (K1107, K3661, and K2734) maintain the significant delay in peduncle senescence observed in the second experiment (Fig 2). The mutant plants for lines K3661 and K1107 are compared to the “Combined WT” of all WT plants from the remaining lines (see further details in methods). N is between 3 and 10. Statistical tests were carried out using the two-sample Wilcoxon test; *, p < 0.05; **, p < 0.01; ***, p < 0.001.


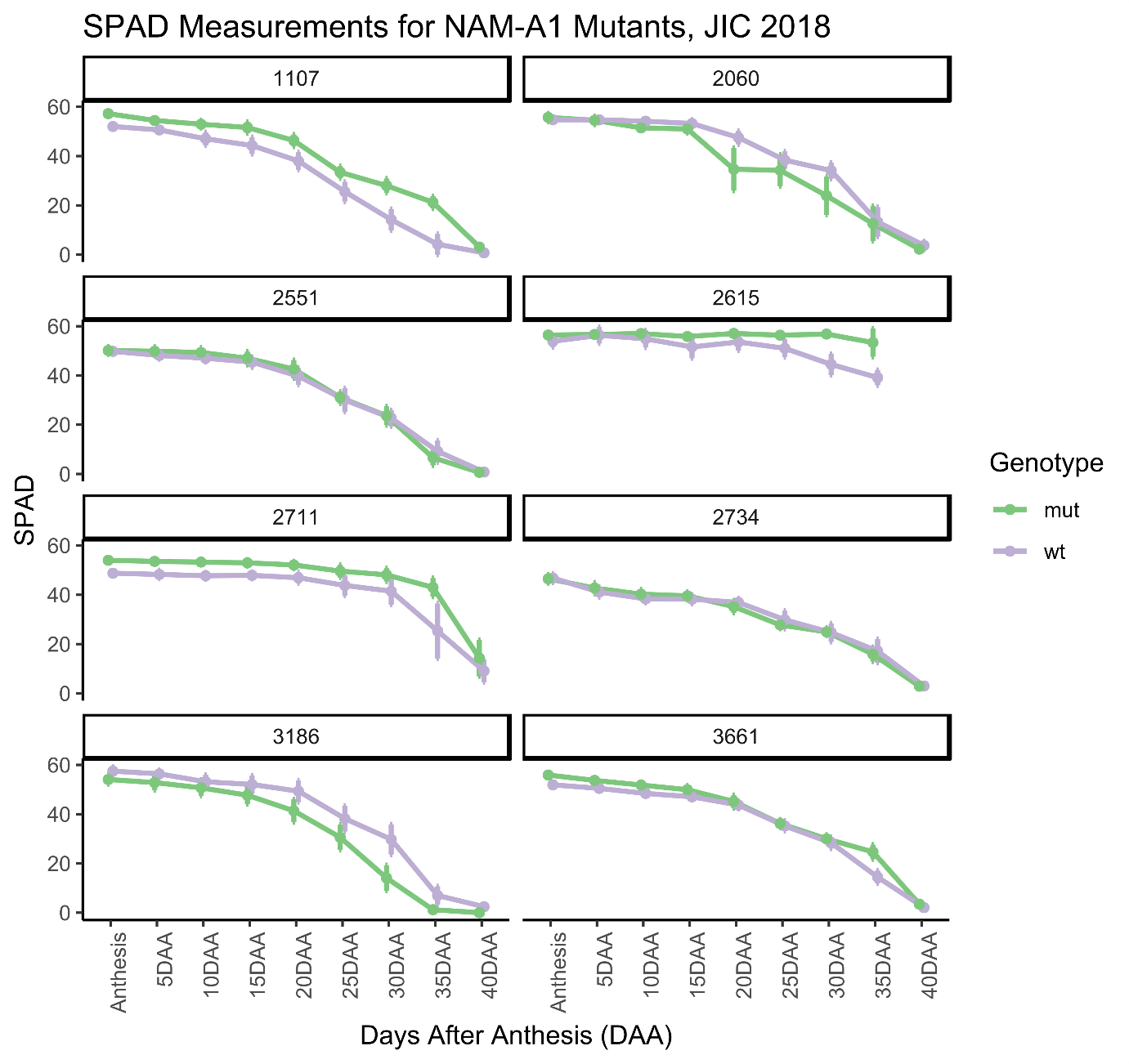


**Figure S3: No significant differences in SPAD measurements are seen for the NAM-A1 TILLING mutants.** SPAD measurements were taken of the primary flag leaf from anthesis to 40 DAA in the glasshouse. Across all timepoints, no significant differences in SPAD units were observed for the mutant plants compared to wild-type for any of the TILLING lines. This correlates with no variation in leaf senescence onset being observed for the lines (Fig 2). N = 5 for all lines, genotypes, and timepoints. Statistical comparisons were carried out using the Kruskal-Wallis Rank Sum test.


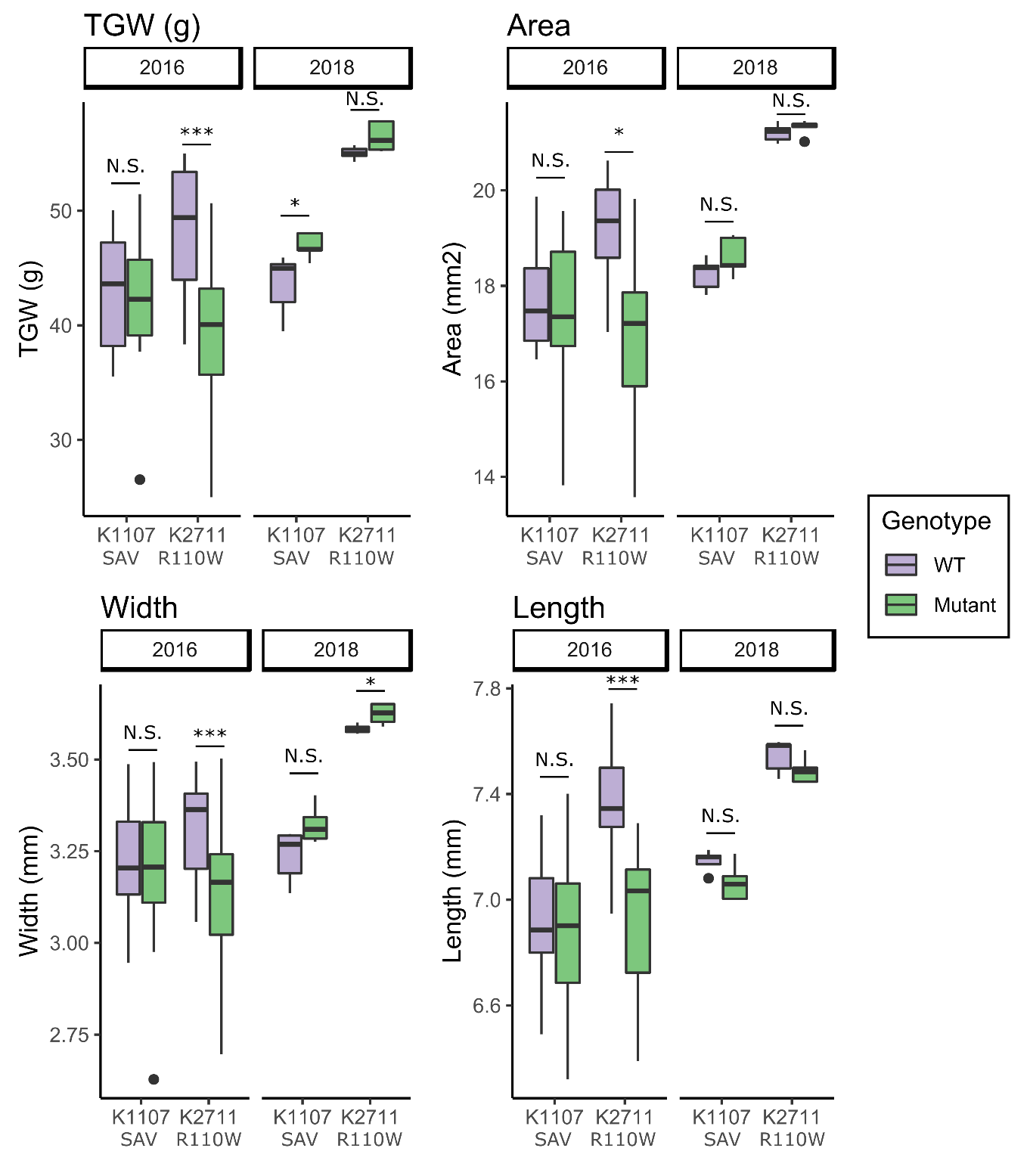


**Figure S4: No consistent variation in grain size parameters is seen in the NAM-A1 mutant lines.** Across two years of field trials, neither the K2711 nor K1107 lines show any consistent variation in grain size or total grain weight (TGW). Variation in grain size and weight for K2711 in 2016 is most likely due to a background mutation co-segregating with the mutant allele that led to a substantial decrease in plant size (data not shown). This variation was lost in 2018, when lines had been selected to remove the co-segregating mutation. Statistical comparisons were carried out using the Kruskal-Wallis Rank Sum test; *, p < 0.05; **, p < 0.01; ***, p < 0.001.


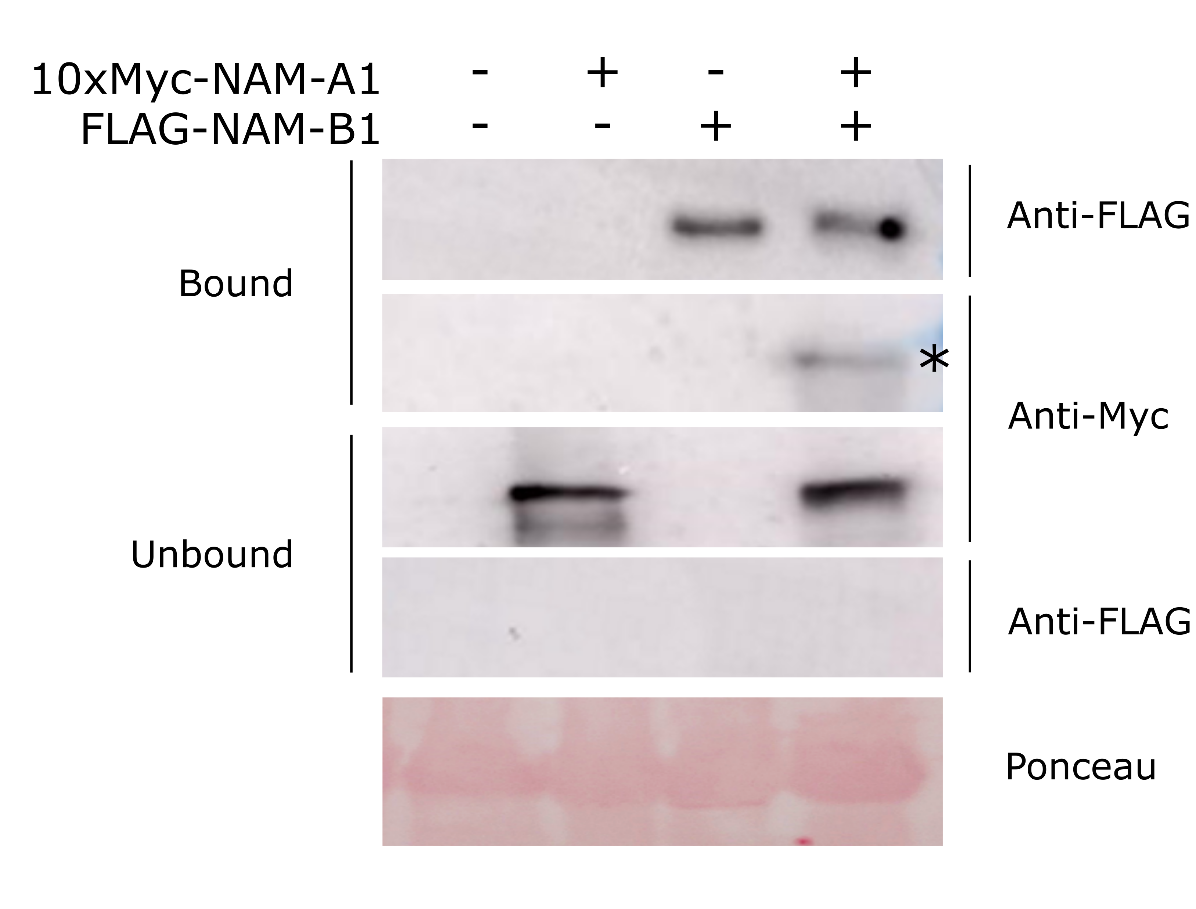


**Figure S5: NAM-A1 interacts with NAM-B1 in a co-immunoprecipitation.** 10xMyc-tagged NAM-A1 is pulled down alongside FLAG-tagged NAM-B1 using anti-FLAG magnetic beads. The asterisk indicates the co-immunoprecipitated 10xMyc-tagged NAM-A1. FLAG-tagged bands are approximately 46 kDa; 10xMyc-tagged bands are approximately 51 kDa. Ponceau stain is of total protein input (Rubisco).


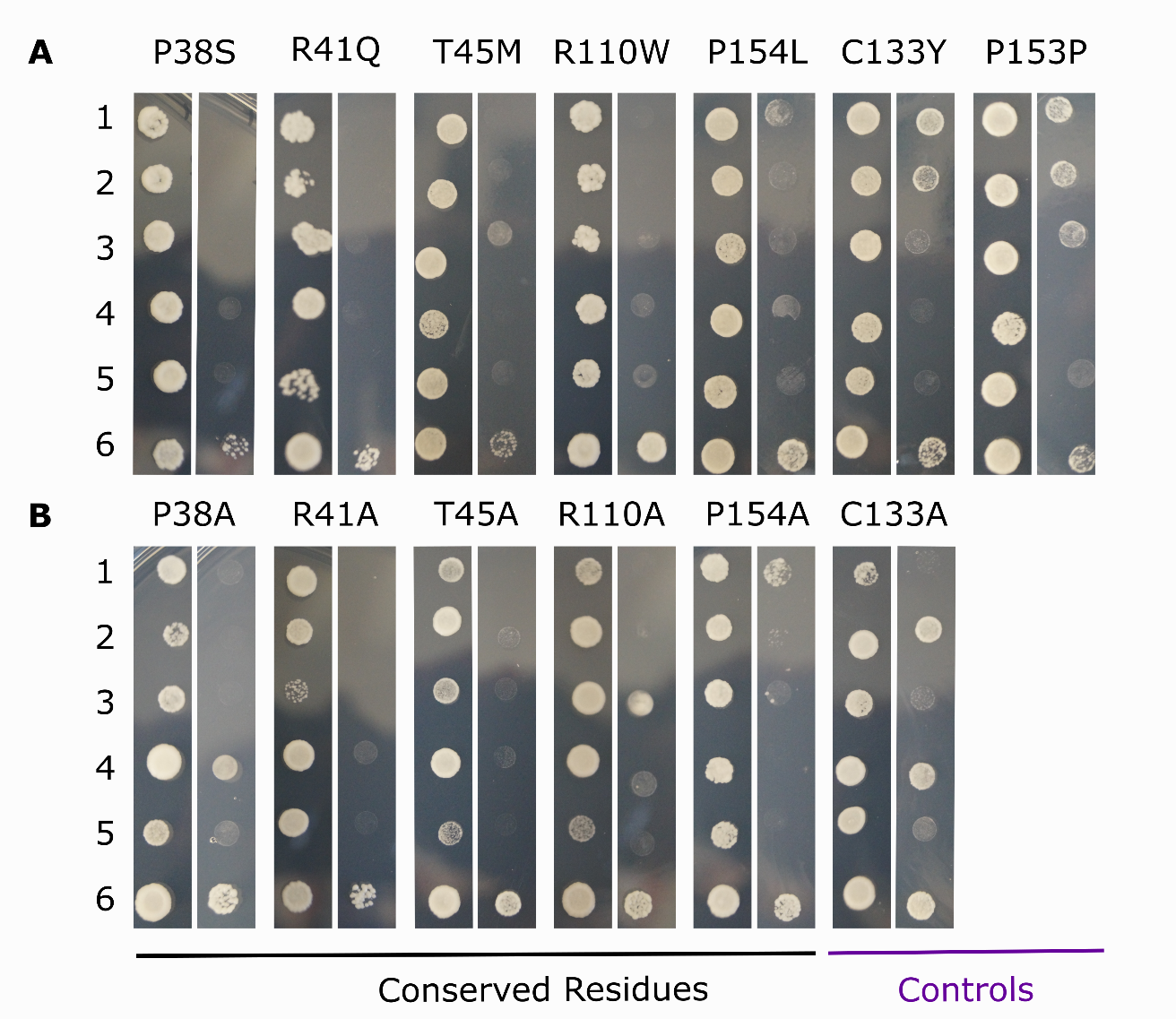


**Figure S6: Mutations in conserved residues of NAM-A1 predominantly lead to loss of protein interaction.** TILLING (A) and alanine (B) mutant alleles of NAM-A1 were tested on control media (left; SC-LT) and selective media (right; SC-LTH + 10mM 3AT). NAM-A1 alleles were co-transformed with the wild-type NAM-B1 allele (1, NAM-A1 in pDEST22; 2, NAM-A1 in pDEST32). The ability of the mutant allele to interact with itself was also tested (3). Relevant controls were carried out for all alleles including NAM-A1:pDEST32 alone (4), NAM-A1:pDEST22 alone (5), and wild-type NAM-A1:pDEST22 with wild-type NAM-B1:pDEST32 (6).


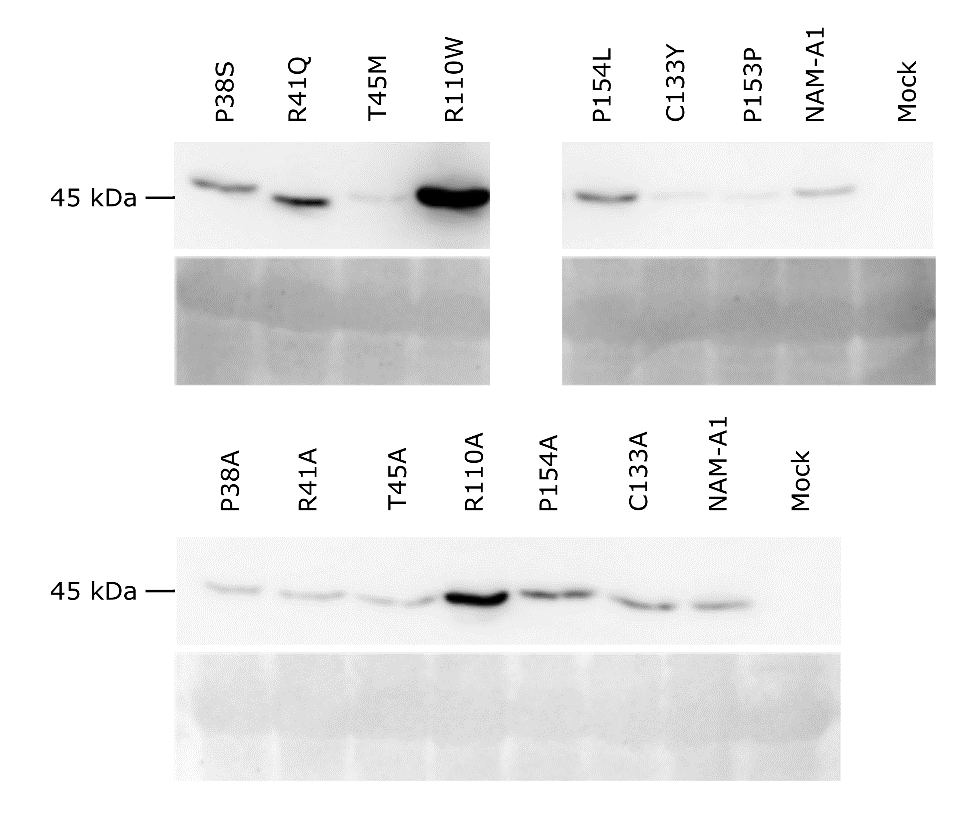


**Figure S7: Expression levels of NAM-A1 mutant alleles does not correlate with observed hypersensitive response.** All constructs showed expression of the FLAG-tagged protein in *N. benthamiana* at 3 dpi. Expression levels were variable but did not correlate with observed cell death responses.

| **Line** | **SNP** | **Codon mutation** | **Het/Hom** | **Consequence** | **cDNA position** | **CDS position** | **Amino acid mutation** | **SIFT** | **JSD Score** | **RefSeqv1 chr6 (bp)** | **Ensembl Link** |
| --- | --- | --- | --- | --- | --- | --- | --- | --- | --- | --- | --- |
| Kronos3661 | C/T | Ccg/Tcg | Hom | Missense | 339 | 112 | P38S | 0 | 0.26 | 77100016 | https://bit.ly/2GLlY71 |
| Kronos2615 | G/A | cGg/cAg | Het | Missense | 349 | 122 | R41Q | 0 | 0.4 | 77100006 | https://bit.ly/2X6gdX3 |
| Kronos2734 | C/T | aCg/aTg | Het | Missense | 361 | 134 | T45M | 0 | 0.46 | 77099994 | https://bit.ly/2S2xUTL |
| Kronos2711 | C/T | Cgg/Tgg | Het | Missense | 555 | 328 | R110W | 0 | 0.27 | 77099581 | https://bit.ly/2X1ZuUW |
| Kronos2551 | G/A | tGc/tAc | Het | Missense | 625 | 398 | C133Y | 0.78 | N/A | 77099511 | https://bit.ly/2Ig556S |
| Kronos2060 | C/T | cCc/cTc | Het | Missense | 688 | 461 | P154L | 0 | 0.17 | 77099448 | https://bit.ly/2SXh2lS |
| Kronos3186 | G/A | ccG/ccA | Het | Synonymous | 686 | 459 | P153P | N/A | 0.16 | 77099450 | https://bit.ly/2N6RCwK |
| Kronos1107 | G/A | N/A | Het | Splice Acceptor Variant | N/A | N/A | N/A | N/A | N/A | 77099212 | https://bit.ly/2GtGqtO |

**Table S1: NAM-A1 mutation location and characterisation.** Details on the individual TILLING SNPs are provided, alongside the heterozygosity of the SNP in the M2 sequencing bulk, predicted consequence, and predicted impact based on conservation and SIFT scores. Links to the variant on EnsemblPlants are also provided.

**Table S2: NAM genes are expressed in the peduncle and stem during reproduction.** Expression data derived from the Wheat Expression Browser (Borrill *et al.* 2016) was obtained for the five functional NAM copies (A1, A2, B2, D1, and D2) from the developmental time course of Azhurnaya (Ramirez-Gonzalez *et al.* 2018). Expression data is given in TPM, ± SEM.

|  |  | *NAM-A1* | *NAM-D1* | *NAM-A2* | *NAM-B2* | *NAM-D2* |
| --- | --- | --- | --- | --- | --- | --- |
| **Timepoint** | **Tissue** | TraesCS6A02G108300 | TraesCS6D02G096300 | TraesCS2A02G201800 | TraesCS2B02G228900 | TraesCS2D02G214100 |
| Ear emergence | Internode #2 | 1.47 ± 0.66 | 1.5 ± 0.92 | 1.26 ± 0.48 | 1.43 ± 0.94 | 1.84 ± 0.9 |
|  | Peduncle | 0.22 ± 0.06 | 0.49 ± 0.32 | 0.15 ± 0.17 | 0 | 0.01 ± 0.02 |
|  | Flag Leaf | 0.46 ± 0.05 | 0.13 ± 0.07 | 0.11 ± 0.11 | 1.19 ± 0.58 | 1.38 ± 0.05 |
| 30% spike | Internode #2 | 0.61 ± 0.37 | 0.55 ± 0.41 | 0.28 ± 0.16 | 0.25 ± 0.19 | 0.87 ± 0.85 |
|  | Peduncle | 0.24 ± 0.06 | 0 | 0.03 ± 0.03 | 0.01 ± 0.01 | 0 |
|  | Flag Leaf | 0.52 ± 0.07 | 0.06 ± 0.01 | 0.1 ± 0.12 | 0.37 ± 0.28 | 0.59 ± 0.28 |
| Milk grain | Internode #2 | 2.57 ± 0.08 | 3.05 ± 0.15 | 5.18 ± 0.96 | 5.42 ± 0.42 | 6.16 ± 0.74 |
|  | Peduncle | 3.56 ± 0.52 | 10.01 ± 1.5 | 11.51 ± 1.66 | 9.79 ± 1.26 | 5.22 ± 1.52 |
|  | Flag Leaf | 1.25 ± 0.46 | 0.3 ± 0.21 | 0.29 ± 0.37 | 4.17 ± 1.99 | 4.12 ± 1.26 |
| Dough | Flag Leaf | 2.92 ± 0.45 | 1.71 ± 0.67 | 1.55 ± 0.65 | 18.79 ± 7.47 | 13.61 ± 3.98 |
| Ripening | Flag Leaf | 9.59 ± 0.93 | 10.03 ± 1.65 | 8.04 ± 0.44 | 48.69 ± 10.66 | 30.25 ± 6.88 |

**Table S3: KASP Primers for NAM-A1 TILLING mutants**

| **Line** | **WT (HEX/VIC)** | **Mut (FAM)** | **Common** |
| --- | --- | --- | --- |
| K3661 | GTGGAACCGGAAGCCCGG | GTGGAACCGGAAGCCCGA | GGCTCGGCGCAAAAAGCA |
| K2734 | CGACCAGCTCCTCGTCCG | CGACCAGCTCCTCGTCCA | AAGCAGCGCGGCATCAGCATG |
| K3741 | CTACTGGAAGGCCACCGG | CTACTGGAAGGCCACCGA | CGCCTTCTTGACGCCGAG |
| K2060 | TTTCTACCGCGGGAAGCCGCC | TTTCTACCGCGGGAAGCCGCT | TGGTGGTGGTGGAGCCAGACA |
| K3186 | TCTTCTACCGCGGGAAGCCG | TCTTCTACCGCGGGAAGCCA | TGGTGGTGGTGGAGCCAGACA |
| K2615 | GGAGCTCCCACCGGGCTTCCG | GGAGCTCCCACCGGGCTTCCA | AAGCAGCGCGGCATCAGCATG |
| K2551 | TGGCCTCGGGGACGGGGTG | TGGCCTCGGGGACGGGGTA | AAGCAGCGCGGCATCAGCATG |
| K2711 | AGCCCGACGTCGCCGCCCG | AGCCCGACGTCGCCGCCCA | TCCATCCATCAGAGAAGGCG |
| K1107 | AGCACCCAGTCGTCCAAC | AGCACCCAGTCGTCCAAT | TCGCACGGTATAGCTAGCAG |

**Table S4: Primers used to amplify the full length and NAC domain fragments (“s”) of wild-type *NAM-A1* and *NAM-B1***

| **Construct** | **Forward** | **Reverse** | **Size of fragment (bp)** |
| --- | --- | --- | --- |
| *NAM-A1* | ATGAGGTCCATGGGCAG | TCAGGGATTCCAGTTCACG | 1227 |
| *NAM-B1* | ATGGGCAGCTCCGACTCA | CGTGAACTGGAATCCCTGA | 1218 |
| *NAM-A1s* | ATGAGGTCCATGGGCAG | TCACTGCTGATCTCCGG | 651 |
| *NAM-B1s* | ATGGGCAGCTCCGACTCA | CCGGCGATCAGCAGTGA | 645 |

**Table S5: Primer sequences for site-directed mutagenesis of the *NAM-A1* alleles.**

|  | **ID** | **Line** | **SNP** | **Primer F** | **Primer R** | **Codon Change** | **Residue Change** |
| --- | --- | --- | --- | --- | --- | --- | --- |
| ***TILLING Mutants*** | SD1_P38S | Kronos3661 | C/T | CGGAGCTCCCATCGGGCTTCCGG | CCGGAAGCCCGATGGGAGCTCCG | Ccg/Tcg | P/S |
|  | SD1_R41Q | Kronos2615 | G /A | TCCCACCGGGCTTCCAGTTCCACCC | GGGTGGAACTGGAAGCCCGGTGGGA | cGg/cAg | R/Q |
|  | SD1_T45M | Kronos2734 | C /T | TTCCACCCGATGGACGAGGAGCTGGT | CGACCAGCTCCTCGTCCATCGGGTG | aCg/aTg | T/M |
|  | SD3_R110W | Kronos2711 | C/T | CGCGGCCGAACTGGGCGGCGACG | CGTCGCCGCCCAGTTCGGCCGCG | Cgg/Tgg | R/W |
|  | SD3_G121D | Kronos3741 | G /A | TACTGGAAGGCCACCGACACGGACAA | TTGTCCGTGTCGGTGGCCTTCCAGTA | gGc/gAc | G/D |
|  | SD4_P154L | Kronos2060 | C/T | AAGCCGCTCAAGGGCCTCAAAACCA | TGGTTTTGAGGCCCTTGAGCGGCTT | cCc/cTc | P/L |
|  | SD4_P153P | Kronos3186 | G/A | CGGGAAGCCGCCCAAGGGCC | TGAGGCCCTTGGGCGGCTTC | ccG/ccA | P |
|  | NC_C133Y | Kronos2551 | G/A | ACGGGGTACGGCCTGGTCCG | AGGCCGTACCCCGTCCCCGA | tGc/tAc | C/Y |
|  | **ID** | **Line** | **SNP** | **Primer F** | **Primer R** | **Codon Change** | **Residue Change** |
| ***Alanine Mutants*** | SD1_P38A | Kronos3661 | C/G | CGGAGCTCCCGTCGGGCTTCCGG | CCGGAAGCCCGCTGGGAGCTCCG | Ccg/Gcg | P/A |
|  | SD1_R41A | Kronos2615 | CG/GC | TCCCACCGGGCTTCGCGTTCCACCC | GGGTGGAACGCGAAGCCCGGTGGGA | CGg/GCg | R/A |
|  | SD1_T45A | Kronos2734 | A/G | TTCCACCCGGCGGACGAGGAGCTGGT | CGACCAGCTCCTCGTCCGCCGGGTG | Acg/Gcg | T/A |
|  | SD3_R110A | Kronos2711 | CG/GC | CGCGGCCGAACGCGGCGGCGACG | CGTCGCCGCCGCGTTCGGCCGCG | CGg/GCg | R/A |
|  | SD3_G121A | Kronos3741 | G/C | TACTGGAAGGCCACCGCCACGGACAA | TTGTCCGTGGCGGTGGCCTTCCAGTA | gGc/gCc | G/A |
|  | SD4_P154A | Kronos2060 | C/G | AAGCCGGCCAAGGGCCTCAAAACCA | TGGTTTTGAGGCCCTTGGCCGGCTT | Ccc/Gcc | P/A |
|  | NC_C133A | Kronos2551 | TG/GC | ACGGGGGCCGGCCTGGTCCG | AGGCCGGCCCCCGTCCCCGA | TGc/GCc | C/A |
