# Supplementary material for: Conserved residues in the wheat (Triticum aestivum) NAM-A1 NAC domain are required for protein binding and when mutated lead to delayed peduncle and flag leaf senescence
Source: BMC Plant Biol. 2019 Sep 18;19:407. doi: 10.1186/s12870-019-2022-5 (PMC6749658; doi:10.1186/s12870-019-2022-5)
Supplement: Supplementary file 3 — Additional file 3: “Gene and Protein Sequences of NAM genes”. This file contains the gene and protein sequences of the full and truncated version of NAM-A1 and NAM-B1 used for cloning and experiments. (DOCX 13 kb) [file 12870_2019_2022_MOESM3_ESM.docx]

**Additional File 3**: CDS and protein sequences of *NAM-A1* and *NAM-B1* used as wild-type controls for yeast 2-hybrid and *N. benthamiana* cell death assays. Subdomains i – v are highlighted in green on the protein sequences.

> TraesCS6A01G108300.2; NAM-A1

CDS:

ATGAGGTCCATGGGCAGCTCCGACTCATCCTCCGGCTCGGCGCAAAAAGCAGCGCGGCATCAGCATGAGCCGCCGCCTCCGCGGCAGCGGGGCTCGGCGCCGGAGCTCCCACCGGGCTTCCGGTTCCACCCGACGGACGAGGAGCTGGTCGTGCACTACCTCAAGAAGAAGGCCGCCAAGGTGCCGCTCCCCGTCACCATCATCGCCGAGGTGGATCTCTACAAGTTCGACCCATGGGAGCTCCCCGAGAAGGCGACCTTCGGGGAGCAGGAGTGGTACTTCTTCAGCCCGCGCGACCGCAAGTACCCCAACGGCGCGCGGCCGAACCGGGCGGCGACGTCGGGCTACTGGAAGGCCACCGGCACGGACAAACCTATCCTGGCCTCGGGGACGGGGTGCGGCCTGGTCCGGGAGAAGCTCGGCGTCAAGAAGGCGCTCGTCTTCTACCGCGGGAAGCCGCCCAAGGGCCTCAAAACCAACTGGATCATGCACGAGTACCGCCTCACCGACGCGTCTGGCTCCACCACCACCAGCCGGCCGCCGCCGCCTGTGACCGGCGGGAGCCGGGCTGCAGCCTCTCTGAGGTTGGACGACTGGGTGCTGTGCCGCATCTACAAGAAGATCAACAAGGCCGCGGCCGGAGATCAGCAGAGGAGCACGGAGTGCGAGGACTCCGTGGAGGACGCGGTCACCGCGTACCCGCTCTATGCCACGGCGGGCATGGCCGGTGCAGGTGCGCATGGCAGCAACTACGCTTCACCTTCACTGCTCCATCATCAGGACAGCCATTTCCTGGAGGGCCTGTTCACAGCAGACGACGCCGGCCTCTCGGCGGGCGCCACCTCGCTGAGCCACCTGGCCGCGGCGGCGAGGGCGAGCCCGGCTCCGACCAAACAGTTTCTCGCCCCGTCGTCTTCAACCCCGTTCAACTGGCTCGATGCGTCACCCGCCGGCATCCTGCCACAGGCAAGGAATTTCCCTGGGTTTAACAGGAGCAGAAACGTCGGCAATATGTCGCTGTCATCGACGGCCGACATGGCTGGCGCGGCCGGCAATGCGGTGAACGCCATGTCCGCATTTATGAATCCTCTCCCCGTGCAAGACGGGACGTACCATCAACACCATGTCATCCTCGGCGCCCCACTGGCGCCAGAGGCTACCACAGGCGGCGCCACCTCTGGTTTCCAGCATCCCGTCCAAGTATCCGGCGTGAACTGGAATCCCTGA

Protein:

MRSMGSSDSSSGSAQKAARHQHEPPPPRQRGSAPELPPGFRFHPTDEELVVHYLKKKAAKVPLPVTIIAEVDLYKFDPWELPEKATFGEQEWYFFSPRDRKYPNGARPNRAATSGYWKATGTDKPILASGTGCGLVREKLGVKKALVFYRGKPPKGLKTNWIMHEYRLTDASGSTTTSRPPPPVTGGSRAAASLRLDDWVLCRIYKKINKAAAGDQQRSTECEDSVEDAVTAYPLYATAGMAGAGAHGSNYASPSLLHHQDSHFLEGLFTADDAGLSAGATSLSHLAAAARASPAPTKQFLAPSSSTPFNWLDASPAGILPQARNFPGFNRSRNVGNMSLSSTADMAGAAGNAVNAMSAFMNPLPVQDGTYHQHHVILGAPLAPEATTGGATSGFQHPVQVSGVNWNP*

> TraesCS6A01G108300.2; NAM-A1 (NAC domain, 1-217)

CDS:

ATGAGGTCCATGGGCAGCTCCGACTCATCCTCCGGCTCGGCGCAAAAAGCAGCGCGGCATCAGCATGAGCCGCCGCCTCCGCGGCAGCGGGGCTCGGCGCCGGAGCTCCCACCGGGCTTCCGGTTCCACCCGACGGACGAGGAGCTGGTCGTGCACTACCTCAAGAAGAAGGCCGCCAAGGTGCCGCTCCCCGTCACCATCATCGCCGAGGTGGATCTCTACAAGTTCGACCCATGGGAGCTCCCCGAGAAGGCGACCTTCGGGGAGCAGGAGTGGTACTTCTTCAGCCCGCGCGACCGCAAGTACCCCAACGGCGCGCGGCCGAACCGGGCGGCGACGTCGGGCTACTGGAAGGCCACCGGCACGGACAAACCTATCCTGGCCTCGGGGACGGGGTGCGGCCTGGTCCGGGAGAAGCTCGGCGTCAAGAAGGCGCTCGTCTTCTACCGCGGGAAGCCGCCCAAGGGCCTCAAAACCAACTGGATCATGCACGAGTACCGCCTCACCGACGCGTCTGGCTCCACCACCACCAGCCGGCCGCCGCCGCCTGTGACCGGCGGGAGCCGGGCTGCAGCCTCTCTGAGGTTGGACGACTGGGTGCTGTGCCGCATCTACAAGAAGATCAACAAGGCCGCGGCCGGAGATCAGCAG

Protein:

MRSMGSSDSSSGSAQKAARHQHEPPPPRQRGSAPELPPGFRFHPTDEELVVHYLKKKAAKVPLPVTIIAEVDLYKFDPWELPEKATFGEQEWYFFSPRDRKYPNGARPNRAATSGYWKATGTDKPILASGTGCGLVREKLGVKKALVFYRGKPPKGLKTNWIMHEYRLTDASGSTTTSRPPPPVTGGSRAAASLRLDDWVLCRIYKKINKAAAGDQQ*

> DQ869673.1; NAM-B1

CDS:

ATGGGCAGCTCCGACTCATCTTCCGGCTCGGCGCAAAAAGCAACGCGGTATCACCATCAGCATCAGCCGCCGCCTCCGCAGCGGGGCTCGGCGCCGGAGCTCCCGCCGGGCTTCCGGTTCCACCCGACGGACGAGGAGCTGGTGGTGCACTACCTCAAGAAGAAGGCCGACAAGGCGCCGCTCCCCGTCAACATCATCGCCGAGGTGGATCTCTACAAGTTCGACCCATGGGAGCTCCCCGAGAAGGCGACCATCGGGGAGCAGGAGTGGTACTTCTTCAGCCCGCGCGACCGCAAGTACCCCAACGGCGCGCGGCCGAACCGGGCGGCGACGTCGGGGTACTGGAAGGCCACCGGCACGGACAAGCCTATCCTGGCCTCGGGGACGGGGTGCGGCCTGGTCCGGGAGAAGCTCGGCGTCAAGAAGGCGCTCGTGTTCTACCGCGGGAAGCCGCCCAAGGGCCTCAAAACCAACTGGATCATGCACGAGTACCGCCTCACCGACGCATCTGGCTCCACCACCGCCACCAACCGACCGCCGCCGGTGACCGGCGGGAGCAGGGCTGCTGCCTCTCTCAGGTTGGACGACTGGGTGCTGTGCCGCATCTACAAGAAGATCAACAAGGCCGCGGCCGGCGATCAGCAGAGGAACACGGAGTGCGAGGACTCCGTGGAGGACGCGGTCACCGCGTACCCGCTCTATGCCACGGCGGGCATGACCGGTGCAGGTGCGCATGGCAGCAACTACGCTTCACCTTCACTGCTCCATCATCAGGACAGCCATTTCCTGGACGGCCTGTTCACAGCAGACGACGCCGGCCTCTCGGCGGGCGCCACCTCGCTGAGCCACCTAGCAGCGGCGGCGAGGGCAAGCCCGGCTCCGACCAAACAGTTTCTCGCCCCGTCGTCTTCGACCCCGTTCAACTGGCTCGATGCGTCACCAGTCGGCATCCTCCCACAGGCAAGGAATTTTCCTGGGTTTAACAGGAGCAGAAACGTCGGCAATATGTCGCTGTCATCGACGGCCGACATGGCTGGCGCAGTGGACAACGGTGGAGGCAATGCGGTGAACGCCATGTCTACCTATCTTCCCGTGCAAGACGGGACGTACCATCAGCAGCATGTCATCCTCGGCGCTCCGCTGGTGCCAGAAGCCGCCGCCGCCACCTCTGGATTCCAGCATCCCGTTCAAATATCCGGCGTGAACTGGAATCCCTGA

Protein:

MGSSDSSSGSAQKATRYHHQHQPPPPQRGSAPELPPGFRFHPTDEELVVHYLKKKADKAPLPVNIIAEVDLYKFDPWELPEKATIGEQEWYFFSPRDRKYPNGARPNRAATSGYWKATGTDKPILASGTGCGLVREKLGVKKALVFYRGKPPKGLKTNWIMHEYRLTDASGSTTATNRPPPVTGGSRAAASLRLDDWVLCRIYKKINKAAAGDQQRNTECEDSVEDAVTAYPLYATAGMTGAGAHGSNYASPSLLHHQDSHFLDGLFTADDAGLSAGATSLSHLAAAARASPAPTKQFLAPSSSTPFNWLDASPVGILPQARNFPGFNRSRNVGNMSLSSTADMAGAVDNGGGNAVNAMSTYLPVQDGTYHQQHVILGAPLVPEAAAATSGFQHPVQISGVNWNP*

> DQ869673.1; NAM-B1 (NAC domain, 1-215)

CDS:

ATGGGCAGCTCCGACTCATCTTCCGGCTCGGCGCAAAAAGCAACGCGGTATCACCATCAGCATCAGCCGCCGCCTCCGCAGCGGGGCTCGGCGCCGGAGCTCCCGCCGGGCTTCCGGTTCCACCCGACGGACGAGGAGCTGGTGGTGCACTACCTCAAGAAGAAGGCCGACAAGGCGCCGCTCCCCGTCAACATCATCGCCGAGGTGGATCTCTACAAGTTCGACCCATGGGAGCTCCCCGAGAAGGCGACCATCGGGGAGCAGGAGTGGTACTTCTTCAGCCCGCGCGACCGCAAGTACCCCAACGGCGCGCGGCCGAACCGGGCGGCGACGTCGGGGTACTGGAAGGCCACCGGCACGGACAAGCCTATCCTGGCCTCGGGGACGGGGTGCGGCCTGGTCCGGGAGAAGCTCGGCGTCAAGAAGGCGCTCGTGTTCTACCGCGGGAAGCCGCCCAAGGGCCTCAAAACCAACTGGATCATGCACGAGTACCGCCTCACCGACGCATCTGGCTCCACCACCGCCACCAACCGACCGCCGCCGGTGACCGGCGGGAGCAGGGCTGCTGCCTCTCTCAGGTTGGACGACTGGGTGCTGTGCCGCATCTACAAGAAGATCAACAAGGCCGCGGCCGGCGATCAGCAGTGA

Protein:

MGSSDSSSGSAQKATRYHHQHQPPPPQRGSAPELPPGFRFHPTDEELVVHYLKKKADKAPLPVNIIAEVDLYKFDPWELPEKATIGEQEWYFFSPRDRKYPNGARPNRAATSGYWKATGTDKPILASGTGCGLVREKLGVKKALVFYRGKPPKGLKTNWIMHEYRLTDASGSTTATNRPPPVTGGSRAAASLRLDDWVLCRIYKKINKAAAGDQQ*
